# Vision Research Literature May Not Represent the Full Intellectual Range of Autism Spectrum Disorder

**DOI:** 10.3389/fnhum.2017.00057

**Published:** 2017-02-14

**Authors:** Alyse C. Brown, Philippe A. Chouinard, Sheila G. Crewther

**Affiliations:** School of Psychology and Public Health, La Trobe UniversityMelbourne, VIC, Australia

**Keywords:** autism spectrum disorders, intelligence quotient, visual perception, DSM 5

## Abstract

Sensory, in particular visual processing is recognized as often perturbed in individuals with Autism Spectrum Disorder (ASD). However, in terms of the literature pertaining to visual processing, individuals in the normal intelligence range (IQ = 90–110) and above, are more frequently represented in study samples than individuals who score below normal in the borderline intellectual disability (ID) (IQ = 71–85) to ID (IQ < 70) ranges. This raises concerns as to whether or not current research is generalizable to a disorder that is often co-morbid with ID. Thus, the aim of this review is to better understand to what extent the current ASD visual processing literature is representative of the entire ASD population as either diagnosed or recognized under DSM-5. Our recalculation of ASD prevalence figures, using the criteria of DSM-5, indicates approximately 40% of the ASD population are likely to be ID although searching of the visual processing literature in ASD up to July 2016 showed that only 20% of papers included the ASD with-ID population. In the published literature, the mean IQ sampled was found to be 104, with about 80% of studies sampling from the 96–115 of the IQ range, highlighting the marked under-representation of the ID and borderline ID sections of the ASD population. We conclude that current understanding of visual processing and perception in ASD is not based on the mean IQ profile of the DSM-5 defined ASD population that now appears to lie within the borderline ID to ID range. Give the importance of the role of vision for the social and cognitive processing in ASD, we recommend accurately representing ASD via greater inclusion of individuals with IQ below 80, in future ASD research.

With the release of the DSM-5 in 2013, previously discrete diagnostic categories such as Asperger's Disorder and Pervasive Developmental Disorder (PDD)—Not Otherwise Specified were collapsed into a single diagnostic category now termed Autism Spectrum Disorder (ASD). Furthermore, sensory abnormalities are now specifically listed in the DSM-5 criteria alongside the more traditionally understood characteristics of impaired social and cognitive abilities. However, there will also be the loss of a subgroup of individuals who previously under DSM-IV would have received an autism spectrum diagnosis, but will now better meet the criteria for a new separate disorder, Social Communication Disorder, distinct from ASD. Thus, such categorization changes will invariably affect the overall accepted prevalence and characteristics of the now reclassified ASD population.

ASD is well accepted as a multi-dimensional disorder with no single cause (Happé et al., 2006) and often involves numerous comorbidities including intellectual development disorder (ID), language disorder and attention-deficit/hyper-activity disorder (Gillberg and Fernell, 2014). These factors contribute to the large amounts of inherent variability within the spectrum beyond the behavioral criteria used to diagnose. Indeed, a feature in need of closer research scrutiny is the contribution of intelligence to the literature relating to social, cognitive and sensory processing of individuals with ASD as currently there is an over-representation of normal (IQ = 90–110) to above (IQ >111) intelligence in samples chosen for most studies (see reviews by Simmons et al., 2009; Philip et al., 2012; Haar et al., 2014; Muth et al., 2014). In particular, the aforementioned reviews raise concerns regarding the generalizability of the current literature to a disorder that has long been accepted as having a high rate of comorbidity with ID (IQ < 70) ((DSM III, IV, 5 Edelson, 2006). Thus, this review examines the range of IQ based sampling that has occurred in the literature associated with visual processing, to demonstrate a trend that is predicted to extend into other ASD research fields (Simmons et al., 2009; Haar et al., 2014).

Since Shah and Frith's seminal paper (1983), a unique pattern of strengths and weakness associated with ASD and intelligence has been recognized. This is most clearly demonstrated by the subtest scores of those with ASD on Wechsler's intelligence scale for children (WISC) (Shah and Frith, 1983). Individuals with ASD exhibit relatively high performance on the Block Design task even as general IQ decreases (Lincoln et al., 1988; Allen et al., 1991; Venter et al., 1992; Shah and Frith, 1993; Happe, 1994; Muth et al., 2014). Furthermore, while language based subtests are predictive of general IQ in typically developing populations, this is not considered to be the case for ASD (Bello et al., 2008; Charman et al., 2011; Muth et al., 2014; Courchesne et al., 2015). Similar comment on the pattern of strengths and weaknesses of visual processing associated with intelligence in ASD is limited due to the predominant use of the ASD without-ID population (see Muth et al., 2014 for comment). For example, a recent meta-analysis on static global/local (whole vs the parts) processing in ASD from Van der Hallen et al. (2015) found that IQ had no influence on performance outcomes. However, given only 9% of ASD studies in the meta-analysis, included individuals with ASD + ID and the fact that studies used only crude IQ groupings (40–70, 70–100, and 100–120), the evidence for IQ not effecting performance outcomes is inadequate. Muth et al. (2014) noted in previously reported results that it only took a small difference in IQ scores between groups to modify research outcomes. Thus, we are arguing that there remains a need to re-examine the intelligence range of the ASD population previously included within studies as another potential factor influencing the inconsistencies concerning generalization of visual processing among individuals with ASD.

The question of variance within the ASD literature pertaining to visual processing is complex and multidimensional. For example, there is little consensus concerning motion processing in the ASD literature. Indeed, within at least the following studies (Koldewyn et al., 2010; Jones et al., 2011), IQ is implicated as an influencing factor. Koldewyn et al. (2010) tested a population with a mean IQ of 107 (*SD* = 16, range = 83–133) while Jones et al. (2011) utilized an IQ population with a mean of 85 (*SD* = 17, range = 52–133) that included 18% with comorbid ASD and ID. Both studies noted exaggerated drops in task performance by the lower IQ scoring ASD participants compared to their IQ matched controls. Performance differences in visual illusion susceptibility have also been noted between mild learning disorder (MLD) groups but not between the groups without MLD (Ropar and Mitchell, 1999). Similarly in a visual evoked potential study examining neural correlates of texture and contour integration, differences were only found in the ASD with-ID sample (Pei et al., 2009). Both of these latter two studies used child samples matched on verbal mental age and reported no effect for chronological age. The studies discussed here leave open the possibility that individuals with ASD who are borderline ID and especially those with ID perform differently to those with ASD but who are within the normal IQ range and above (Muth et al., 2014). This is likely to be either because of the ID itself or because of the combination of co-morbid problems as has previously been noted by Braddick and colleagues (Braddick et al., 2003; Braddick and Atkinson, 2013). In brief, it is difficult to disentangle the factors effecting and characterizing visual perception in ASD with-ID both because of the common practice of selection bias and differences in ability to attend and comprehend verbally based task prescriptions (Williamson et al., 2005). Thus the following theoretical questions remain (*i*.) are the visual processing differences in ASD with-ID consistent? or (*ii*.) are the visual deficits an additional characteristic only in those with ID co-morbid for ASD? and /or (*iii*.) do these individuals actually process differently to idiopathic ID without ASD?

With the verdict unclear as to the nature of visual processing in individuals with comorbid ASD and ID, there is a critical need to determine to what extent the current ASD visual processing literature is representative of the entire spectrum of the ASD population as a whole. Thus, in this review, we have explored these questions by first establishing the relative estimated prevalence of ASD with-ID and other IQ ranges in the ASD literature as of 2016. We then report the sampling of IQ ranges within currently available research literature dealing with visual processing and determine if the current literature is in fact representative of visual processing in the current ASD population that is recognized and defined by DSM-5. Finally, we discuss possible solutions to the operational confounds of testing individuals within the borderline ID, and ID IQ ranges, with the aim of extending research into the relationships between ASD visual processing and intelligence and core characteristic of social communication impairment in ASD.

## Prevalence of IQ ranges in ASD

Pre DSM-5, autism was clinically considered a disorder predominantly characterized by a comorbid diagnosis of ID (Edelson, 2006). Indeed, the DSM-III reported in 1987 the prevalence of ID individuals in the autism population to be 70–75% (American Psychiatric Association., and American Psychiatric Association Work Group to Revise DSM-III., 1987). Furthermore in 2001 a PDD prevalence study, using DSM-IV criteria, showed that the percentage of individuals with ID in the autism subgroup remained at 70% (Chakrabarti and Fombonne, 2001). However, the IQ prevalence figures have changed considerably since the DSM-5 in 2013, with the diagnostic criteria for ASD now including groups not previously associated with ID, i.e., Asperger Disorder and PDD. Indeed, under the new DSM-5 criteria, some individuals previously diagnosed with Asperger Syndrome and PDD under the DSM-IV would not meet the criteria of “deficits in social communication and interactions” and “deficits in verbal and nonverbal communication” (Mattila et al., 2011). For those with Asperger Syndrome and PDD diagnosed under DSM-IV criteria, the diagnosis remains due to the grandfather clause, meaning any ensuing reduction in individuals without ID will take time to impact overall IQ prevalence statistics.

There are several recent studies exploring the current prevalence of IQ in the ASD population as outlined by the DSM-5. Firstly, in 2014, the United States Centre for Disease Control and Human Services (CDC) reported the following IQ distributions in people with ASD as defined by the DSM-5: 31% in the ID range (IQ < 70), 23% in the borderline ID range (IQ = 71–85), and 46% in the range above (IQ > 85). Other studies (Chakrabarti and Fombonne, 2001; Icasiano et al., 2004; Baird et al., 2006; Charman et al., 2011) that included DSM-IV diagnosed autism, and PDD and Asperger Syndrome populations that are now recognized by the DSM-5 as ASD report ASD with-ID prevalence figures between 25 and 46%. An unweighted average of the following studies (Chakrabarti and Fombonne, 2001; Icasiano et al., 2004; Baird et al., 2006; Charman et al., 2011; Wingate et al., 2014) was used to calculate a new ASD with-ID prevalence, this new figure came out at ~42%. This post DSM-5 estimated prevalence of ASD with-ID is substantially lower than the DSM-III and DSM –IV previously quoted prevalence of 70–75%. By deduction approximately 60% of those with ASD have an IQ of above 70.

Mean IQ for those meeting DSM-5 diagnostic criteria for ASD was reported as 69 in a sample of 158 ASD participants who were diagnosed with the ICD-10 (Baird et al., 2006). The ICD-10 diagnoses are reported to be comparable to the DSM-IV criteria for autism, and close to DSM-5 if one includes Asperger's Syndrome and PDD (Volkmar et al., 1994; Sponheim, 1996). Furthermore, the American Wingate et al. (2014) reported the ASD IQ mean to be between *M* = 75 and 85. These data show the typical ASD IQ score to lie within the borderline ID and ID range. These new figures surrounding the prevalence of IQ will now be used in this review to judge the representative nature of IQ samples in the ASD literature.

## IQ sampling in literature pertaining to visual processing

In determining to what extent the ASD with-ID population has been excluded from visual research, we carried out two systematic literature searches that looked for key terms in papers titles and abstracts. The first search specified an ASD without-ID population and the second specified use of an ASD with-ID population. Constant in both searches were the grouped search terms associated with ASD, and with visual processing. We searched the PsycINFO database from its inception until July 2016 and the PubMed databases up to the same dates. Both search engines were checked for samples indicating without-ID and with-ID groups, and were limited to the English language and to experimental papers in peer reviewed journals. Infant studies (birth to 3 year) were excluded as measures of intelligence are less reliable for this demographic. The search results for the combined terms of ASD, visual processing and without-ID found 145 studies whereas in the corresponding ASD with-ID search, 32 studies were identified (see Table 1 for summary of results; see Table 1 and Table 2 in Supplementary Materials for extended search terms and list of search results).

**Table 1:** **Summary of two systematic literature searches for ASD with-ID (Search 1) and ASD without-ID (Search 2) in the ASD visual processing literature**.

**Categories**	**Category search**	**Manual removal**	**Total**
**SEARCH 1**			
With-ID	27,310		
ASD	36,840		
Visual processing	757,770		
Combined categories	32	19	14
**SEARCH 2**			
Without-ID	31,242		
ASD	36,840		
Visual processing	757,770		
Combined categories	145	67	79

*Reporting the number of studies found at each stage of the search*.

The results from both searches were then manually sorted and papers not fitting the criteria were removed. This left only 14 ASD visual processing studies present in the with-ID search results compared with 79 studies in without-ID search results. These two searches give a gross overview of the sampling trends that are present in this literature and indicate that only 20% of visual research relating to individuals with DSM-5 defined ASD, include individuals with a comorbid diagnoses of ID. That is our searches indicate that 80% of the vision research associated with ASD is representative of less than 60% of the appropriate population, i.e., those with ASD without ID while the ASD with ID group who we argue currently represent 42% of the ASD population, have not been adequately considered.

## WISC/WISC IQ sampling in literature pertaining to visual processing

**Figure 1:**
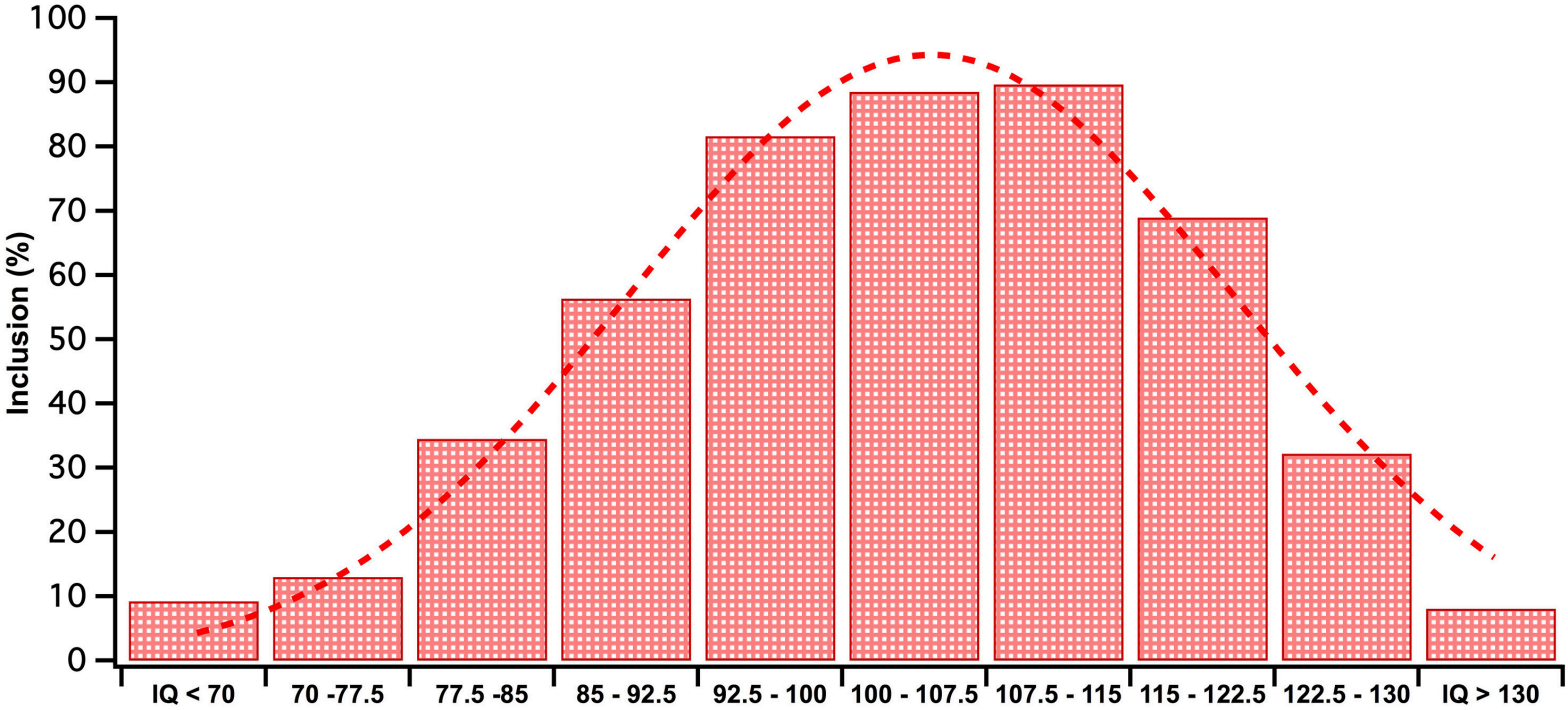
**Given that mean and SD were available for all studies, each data set was modeled as a flat distribution within the range (M−1SD, M+1SD)**. The WISC IQ scores included in studies 68 percentile range was frequency tallied into IQ bins. The frequencies in each IQ bin were converted to inclusion percentages out of total studies (*n* = 87) in the analysis. The RED bars depict these percentages. This data has been fitted with a red Gaussian curve.

Having obtained an initial understanding of what IQ has been sampled in this literature, we then attempted to obtain a more detailed breakdown. The terms ASD without-ID and ASD with-ID encompasses broad ranges of IQ scores. To acquire a more precise understanding of sampled IQ range, the 68% percentile range of ASD IQ scores were collected from studies by using the reported means and standard deviations. Wechsler's child and adult intelligence scales (WAIS, WISC) (Wechsler, 2003, 2014) was chosen for this analysis for scale consistency and because of the frequency with which Wechsler's measure has been used in the literature compared to other IQ measures. Both the full and abbreviated versions of Wechsler's intelligence tests were included. A third search was conducted targeting ASD visual processing literature that utilized the WAIS/WISC. This search found 59 additional studies to the 28 found in the previous searches. In total, these 87 studies were used to conduct a frequency analysis of different IQ score ranges (see Figure 1 for results).

As shown in Figure 1, over 80% of studies we searched included IQ scores in the range between 96 and 115, emphasizing that most of our knowledge on ASD visual processing comes from the slightly higher end of the normal IQ range (IQ = 90–110) and above. The spread of sampled IQ scores in Figure 1 resembles a unimodal distribution with a mean IQ score of 104. This is far from an accurate reflection of the ASD IQ spread reported by the American Wingate et al. (2014) as a ASD IQ mean between *M* discrepancy between the typical IQ profile of ASD and the population on which visual research has been conducted.

Figure 1 indicates that the current rate of inclusion of the ID range for studies that use Wechsler's tests is 9%, a number substantially lower than our calculated prevalence average of ~40% (Chakrabarti and Fombonne, 2001; Icasiano et al., 2004; Baird et al., 2006; Charman et al., 2011; Wingate et al., 2014). These numbers may involve some biased under-reporting of studies including the ID range as our criteria was studies using Wechsler's test, and excluded those studies where IQ was measured using non-verbal tests such as the Raven's Progressive Matrices (Raven, 1938) or behavioral measures e.g., Vineland adaptive behavior scales (Sparrow et al., 1984). Such non-verbal IQ measures have often been employed in studies with individuals who were unable to adequately complete the Wechsler's more verbally based sub-tests on which they often achieve a floor score (Bello et al., 2008; Courchesne et al., 2015). Prevalence of borderline ID in the ASD population is rarely documented though the recent CDC report nominated the prevalence of borderline ID as 24%. This number is lower than illustrated in Figure 1, though it is interesting to note that the majority of studies including the borderline part of the IQ spectrum, came from the 77.5 to 85% upper end of this population. This suggests that the lower IQ grouping 70–77.5 range have been neglected with only a 13% inclusion rate. We would argue that the ASD with-ID inclusion rate was better captured in our systematic literature search that indicated that 20% of papers include the ID range, while the without ID sampling trends has been more effectively captured in Figure 1.

Overall consideration of both methods of reviewing the sampled IQ within the literature demonstrates a mismatch between inclusion rate of some of the borderline ID (IQ 70–80) and all the ID (IQ < 70) populations compared with their general prevalence is substantial. Thus, we are unsure if the current literature with its preponderance of studies using ASD samples with IQ in the normal range (90–110) can be generalized to the typical level of intelligence found in the ASD population that has been re-established here as being predominantly below an IQ of 85. The current situation suggests that greater effort must be made to include individuals with ID and those with IQ levels < 80, in studies of visual processing associated with ASD so that the impairments in social and cognitive processing that characterize and define ASD and are also underpinned by visual information and visual processing, can be better understood in terms of the general ASD population.

Reluctance to test individuals who are below 80 in IQ is presumably a practical stance as the data collected from these individuals are often hard to obtain, and often close to floor level performance. The data from these individuals is therefore less reliable. Indeed, results can be affected by many external factors such as poor comprehension of task instructions, straying of attention, increased rates of button press errors, less working memory (Braddick et al., 2003). However, visual processing research in infant populations has demonstrated effectively that alternative study designs and technologies do not have to rely on verbal instruction and/or sustained attention (Martin et al., 1999; Roder et al., 2000; de Haan et al., 2002). Recording techniques such as electrophysiology and MEG can inform source localization and trajectory of visual information and processing without cognition needing to be a factor (Gage et al., 2003; Wilson et al., 2007). Eye tracking systems can inform on differences in patterns of eye movements and deployment of attention measured as time to activate saccades and accuracy of shifts in attention and duration of fixation, visual search styles as well as where attention is being directed (Falck-Ytter et al., 2015). With simple task designs and the use of recording techniques, individuals with ID would no longer need to be excluded from research.

## Author contributions

AB: Conducted the research that went into this review and wrote the review; SC: Is the primary supervisor of AB and provided guidance and significant input into the writing process; PC: Also a supervisor of AB provided guidance and significant input into the writing process.

### Conflict of interest statement

The authors declare that the research was conducted in the absence of any commercial or financial relationships that could be construed as a potential conflict of interest.
